# Subacromial impingement syndrome: a survey of Italian physiotherapists and orthopaedics on diagnostic strategies and management modalities

**DOI:** 10.1186/s40945-020-00087-7

**Published:** 2020-09-02

**Authors:** Fabrizio Brindisino, Diego Ristori, Mariangela Lorusso, Simone Miele, Leonardo Pellicciari, Giacomo Rossettini, Francesca Bonetti, John Duane Heick, Marco Testa

**Affiliations:** 1 Department of Medicine and Health Science “Vincenzo Tiberio”, University of Molise C/da Tappino c/o Cardarelli Hospital, 86100 Campobasso, Italy; 2 Department of Clinical Sciences and Translational Medicine, Tor Vergata University of Rome, Rome, Italy; 3 Department of Neuroscience, Rehabilitation, Ophtalmology, Genetics, Maternal and Child Health – University of Genova – Campus of Savona, Savona, Italy; 4 Neurorehabilitation Research Laboratory, IRCCS San Raffaele Pisana, Rome, Italy; 5 Department of Physical Therapy and Athletic Training, Northern Arizona University Flagstaff, Flagstaff, AZ USA

**Keywords:** Shoulder impingement syndrome, Orthopaedic manipulative physical therapists, Orthopaedic surgeons, Italian survey, Shoulder pain

## Abstract

**Background and aim:**

The subacromial impingement syndrome (SIS) represents a common cause of disability in approximately 74% of patients with Shoulder Pain (SP). Even if contemporary research suggests that this mechanism is not (always) the dominant driver in SP, SIS is still a source of debate among scholars and clinicians. From a clinical point of view, evidence has suggested that clinicians can use both medical and physiotherapy approaches as effective methods to treat SIS.

This survey aims to investigate models of management of patients with SIS in a sample of Italian physiotherapist specialists (Orthopaedic Manipulative Physical Therapists, −OMPTs-) and orthopaedic surgeons.

**Materials and methods:**

An online survey with 29-item questionnaire was administered to assess the knowledge of OMPTs and orthopaedic surgeons about: a) strategies of clinical examination; b) the role of imaging in the diagnostic process; c) the physiotherapy management; and d) the pharmacological and surgical management in patients with SIS.

**Results:**

Six-hundred and twenty-nine respondents completed the survey (511 OMPTs (79.97%) and 128 orthopaedic surgeons (20.03%)). Ninety-two percent (*n* = 470) of the OMPTs and 80.5% (*n* = 103) of orthopaedic surgeons stated that in patients with SIS, a combination of diagnostic tests produced better accuracy (*p* = < 0.001). Twenty point seven % of OMPTs (*n* = 106) and 4.7% of orthopaedic surgeon (*n* = 6) stated that the Lift off was the most specific test (*p* = < 0.001). Four-hundred-and-twenty-four OMPTs (83%) and 40 orthopaedic surgeons (31.3%) answered that the gold standard for diagnosis of a patient with SIS are history and clinical examination (*p* < 0.001).

**Conclusion:**

OMPTs and orthopaedic surgeons approach patients with SIS differently during both the assessment and the treatment. OMPTs appear to be appropriate in planning and managing clinical examination and therapeutic strategies to use with patients with SIS.

## Background

Shoulder pain (SP) is the third most common musculoskeletal disorder in the general population for which medical care is sought [6, 51]. SP can significantly influence the patient’s ability to work, as well as to perform daily life and recreational activities [68].

The prevalence of SP is estimated to range from 19 to 31% for monthly prevalence; from 5 to 47% for annual prevalence; and from 7 to 67% for lifetime prevalence [50]. The resolution of symptoms (e.g., pain, disability) may be gradual, thus resulting in a high relapse rate. For example, authors have noted that 25% of patients report a previous episode of SP and between 40 to 50% of patients report persistent pain at 12-month follow-up [42]. The annual incidence of SP varies according to the age of the patient: 0.9% between 31 and 35 years; 2.5% between 42 and 46 years; 1.1% between 56 and 60 years; and 1.6% between 70 and 74 years [51, 56].

The Cochrane revision of Hanchard, Lenza, Handoll and Takwoingi [36] identifies subacromial impingement syndrome (SIS) as a common cause of disability without joint stiffness, which occurs in approximately 74% of patients with SP. The pathoanatomical construct of SIS is debated at the international level among scholars and clinicians [7].

The most adopted classification of SIS has been based on localizing the soft tissue entrapment [23]. This classification differentiates internal causes - postero-superior impingement [72] and antero-superior impingement [30] -, from those occurring externally - “outlet impingement” [54]-, such as acromial spurs or uncinated acromion morphology causing extrinsic compression of the subacromial bursae and abrasion of the rotator cuff [28]. Subsequently, Lewis [45] has explained SIS as a consequence of an underlying loss of strength of the rotator cuff, which leads to an alteration of the centered position of the humerus onto the glenoid fossa [46]. Thus, it may be possible that patients presenting with a rotator cuff unable to perform its main function, are in the early stage of SIS [45, 49].

As a consequence of this international debate, SIS has been labelled as a clinical entity, rather than a diagnostic one, responsible for several functional and tissue alterations (e.g., rotator cuff lesions) [36, 41, 52]. Thus, impingement has been defined as an umbrella term for a range of signs and symptoms that are typically seen in patients with SP [37]. The current diagnostic process has been influenced by this concept and the importance of anamnesis and clinical examination in evaluating patients with SIS mirrors this approach. The evolution of knowledge through evidence has influenced clinicians’ understanding in terms of signs and symptoms revealing SIS, as well as orthopaedic tests needed to assess the patient with SP. In fact, in addition to the controversial etiology and the multiplicity of structures and mechanisms that may be involved in SIS, there is also a lack of evidence to support the mechanical construct of physical tests for the diagnosis of SIS. Despite their common use in physical examination as provocative tools [47], their effectiveness in identifying (and isolating) the different structures responsible for the patient’s symptom or replicating a single pathological mechanism has been questioned [7, 37]. Evidence has also influenced the use of imaging for the assessment of the patient with SIS - e.g., Ultrasound (US), Magnetic Resonance Imaging (MRI), Magnetic resonance Arthrography (MRA) - [7, 62].

From a clinical point of view, authors have suggested that clinicians can use both medical and physiotherapy approaches as effective methods to treat SIS. Several physiotherapy treatments for SIS are proposed in the literature, including electrotherapy (e.g., laser, US, heat, shock waves) and taping techniques, as well as manual therapy and therapeutic exercises [18, 20, 55, 73]. Medical treatments for SIS range from pharmaceutical treatments (e.g., Non-Steroidal Anti Inflammatory Drugs – NSAID- and corticosteroids injections) to surgical approaches (e.g., arthroscopic subacromial decompression, open subacromial decompression, arthroscopic bursectomy, acromioplasty performed with radio frequency therapy, injections of platelet and leukocyte enriched gel) [18, 19, 25, 29, 64].

Since there is a wide range of approaches for patients with SP related to SIS, some countries (e.g., United Kingdom, Germany, Netherlands) have conducted surveys aimed at investigating if specific clinicians’ assessment and interventions for patients with SP follow evidence-based practice [9, 48, 58, 59, 67].

Given the lack of data in Italy regarding how specific clinicians assess and treat patients with SIS, this survey aims to investigate models of management of patients with SIS by Italian physiotherapist specialists (Orthopaedic Manipulative Physical Therapists, −-OMPTs-) and orthopaedic surgeons. These two categories have been chosen specifically because they include clinicians who routinely manage patients with SIS in Italy as well as in Europe [16]. A sub category of Italian physiotherapists –OMPTs-, specialized physiotherapists with musculoskeletal training were investigated because evidence supports that providers with advanced competencies are more likely to follow evidence-based practice in their clinical practice [8].

## Materials and methods

### Design

A quantitative exploratory web-based cross-sectional survey was conducted that follows the CHERRIES guidelines [24] and STROBE guidelines [71]. The survey was administered between May and July 2019. Ethical approval was obtained from the Ethics Committee of Lecce (Italy) (Protocol number 33, approved on 06/06/2019). All the study-related procedures were performed according to the principles of the declaration of Helsinki [1].

### Participants and setting

The target populations were Italian physical therapists specialized in musculoskeletal disorder rehabilitation (Orthopaedic Manipulative Physical Therapists -OMPTs-) and orthopaedic surgeons.

Participation was voluntary and no incentives were offered to participants in this survey. University Master courses are training courses required for specialization as OMPTs and are based upon the standards established by IFOMPT (International Federation of Orthopaedic Manipulative Physical Therapists) [5]. The sample of OMPTs was obtained from the databases of the following universities: a) Masters in Rehabilitation of Musculoskeletal Disorders (MRDM) at Genoa University (*n* = 1300); b) Masters in Manual Therapy applied to Physiotherapy at Roma Tor Vergata University (*n* = 140); c) Masters in Manual Therapy and Musculoskeletal Rehabilitation at Padova University (*n* = 98); and d) Masters in Musculoskeletal Physiotherapy, Manual Therapy and Therapeutic Exercise at Bologna University (*n* = 32).

The sample of orthopaedic surgeons was identified from the databases of the 3 most representative Italian orthopaedic associations: a) SICSeG (Società Italiana Chirurgia di Spalla E Gomito) (*n* = 152); b) SIA (Società Italiana di Artroscopia) (*n* = 1470); c) and SIGASCOT (Società Italiana Ginocchio Artroscopia Sport Cartilagine e Tecnologie Ortopediche) (*n* = 1574) that encompasses surgeons specialized in shoulder and elbow surgery.

Clinicians were included if they: a) had a valid e-mail account, b) understood Italian; and c) were working as clinicians in Italy at the time of the survey. Previous surveys [9, 48, 58, 59, 67] reported a response rate ranging from 3% [67] to 21% [59]. Pieters et al. [58] recruited a sample of 505 subjects, Bury and Littlewood [9] 191 subjects, Littlewood, Lowe and Moore [48] 110 respondents, Pribicevic, Pollard and Bonello [59] 112/1037 with a response rate of 21% and Struyf, de Hertogh, Gulinck and Nijs [67] 119/3877 had a response rate of 3%. Taken all together, these previous surveys suggested that the potential response rate would range from 3.0 to 21.0%.

### Questionnaire development

The questions within the survey were derived from systematic reviews and meta-analysis to focus on patients with SIS and particularly on:

- clinical examination [22, 31, 36, 38, 43, 53];
- the role of imaging in the diagnostic process [44, 57, 62];
- the physiotherapists’ management [15, 33, 34, 65, 70];
- and the surgical and pharmacological management of patients with SIS [18, 32, 66].

The questions were critically evaluated for face validity [14] by 6 experts with extensive experience in shoulder diseases (two orthopaedic surgeons and four physical therapists -DR, FB, SM, ML-). These experts worked independently on the survey questions and agreed on the final edited questions by providing feedback on content accuracy, wording, question order and survey structure. When full agreement was achieved, a preliminary version of the survey, consisting of 29 questions (9 demographic questions and 20 technical questions), was piloted to 15 OMPTs (*n* = 5 North; *n* = 5 Center; and *n* = 5 South of Italy) and 15 orthopaedic surgeons (=5 North; *n* = 5 Center; and *n* = 5 South of Italy) aimed at increasing the content validity of the questions.

Moreover, the experts interviewed the sample of 15 OMPTs and orthopaedic surgeons to understand if the questions needed further clarity or if there were any confusing words that required further explanation. Based on their feedback no further edits were necessary. The average time needed to complete the survey was approximately 12.50 min which was advantageous as these clinicians are busy and are potentially unlikely to complete a survey that takes too much time [26]. The final version of the survey consisted of 29 questions (Appendix) that allowed for one answer per question.

In section (A), 9 multiple-choice questions were included to collect the following descriptive data: profession; experience in years; working environment; working field; working status; age; sex; geographic zone of work; and patients with a SIS diagnosis for more than 1 month were investigated.

Section (B) was composed of 20 multiple-choice questions focused on: special tests (5 questions); imaging techniques (4 questions); assessment of patients with SIS (4 questions); physical therapy (4 questions); pharmacological/surgical management of the patient with SIS (2 questions); and outcome measure collection (1 question).

### Data collection

SurveyMonkey (Survey-Monkey, Palo Alto, California, www.surveymonkey.com) was used to collect data and was administered between April 28th, 2019 and June 25th, 2019. The time frame for data collection was considered adequate in accordance with previous surveys conducted on SIS where at the end of the 2 months there were no further requests to complete the survey [48].

Potential participants were initially invited to participate in the survey via email that outlined: a) the aim of the study; b) data handling (anonymity); c) the informed consent statement; and d) the invitation to complete the questionnaire.

Respondents provided consent to participate in the survey by clicking on the survey link. To encourage participation two email reminders were sent at both 4 and 6 weeks after the initial invite to participate in the survey.

When completing the survey, respondents were able to review and modify their responses using a back button before submitting their answers. After submission of the survey, respondents were unable to edit their answers. For data analysis, answers were downloaded and stored to a password protected and encrypted computer. Only the statistician (LP) accessed and analysed the data collected from the surveys. All data were de-identified (name and email address) to maintain confidentiality and data protection [14].

### Data analysis

Descriptive statistics were created by Survey Monkey and downloaded into Excel 2016 (Microsoft Corp. Redmond, WA, USA). Descriptive statistics (mean, standard deviation) were used for continuous variables, while absolute frequencies and percentages were applied to dichotomous, nominal, and ordinal variables. These variables were grouped by demographical data to define the sample recruited. OMPTs and orthopaedic surgeons were separated. Chi-squared tests were completed using SPSS, version 24 (IBM Corp., Armonk, NY, USA) to investigate differences in the responses between the two categories.

## Results

In total, 639 respondents completed the survey with 511 OMPTs (79.97%) and 128 orthopaedic surgeons (20.03%) finalizing the questionnaire to be used in data analysis.

The majority of respondents were male (OMPTs: *n* = 332; 65.0%; orthopaedic surgeons: *n* = 115; 89.8%). The most representative age for OMPTs was < 30 years old (*n* = 281; 55.0%), while orthopaedic surgeons were between 30 and 40 (*n* = 44; 34.4%). The majority of respondents worked in Northern Italy (OMPTs *n* = 316; 61.8%; orthopaedic surgeons *n* = 66; 51.6%) and in the musculoskeletal field (OMPTs *n* = 460; 90.0%; orthopaedic surgeons *n* = 128; 100%). Most OMPTs worked in private practice (*n* = 387; 75.7%) while most orthopaedic surgeons were employees in the National Health System (*n* = 82; 64.1%).

The majority of OMPTs had worked for less than 5 years (*n* = 227; 44%) in a private setting (*n* = 423; 82.8%) and normally see less than 5 patients with SIS per month (*n* = 250; 48.9%). On the other hand, the majority of orthopaedic surgeons had worked for more than 15 years (*n* = 61; 47.7%) in a hospital (*n* = 94; 73.4%) and normally see 5 to 10 patients with SIS per month (*n* = 39; 30.5%). The respondents’ demographics are described in Table 1.

**Table 1:** Demographical Data

Variables	Samples		
	Total (***n*** = 639)	OMPTs (***n*** = 511)	Orthopaedic surgeon (***n*** = 128)
Gender			
Male	447 (70.0%)	332 (65.0%)	115 (89.8%)
Female	192 (30.0%)	179 (35.0%)	13 (10.2%)
Age (Years)			
< 30	283 (44.3%)	281 (55.0%)	2 (1,6%)
> 50	52 (8.1%)	10 (2.0%)	42 (32.8%)
30–40	232 (36.3%)	188 (36.8%)	44 (34.4%)
40–50	72 (11.3%)	32 (6.3%)	40 (31.3%)
Years of Clinical Practice			
< 5	251 (39.3%)	227 (44%)	24 (18.8%)
5–10	192 (30.0%)	172 (33.7%)	20 (15.6%)
11–15	96 (15.0%)	73 (14.3%)	23 (18.0%)
> 15	100 (15.6%)	39 (7.6%)	61 (47.7%)
Workplace			
Private practice	456 (71.4%)	423 (82.8%)	33 (25.8%)
Hospital	147 (23.0%)	53 (10.4%)	94 (73.4%)
Residential care (nursing home)	36 (5.6%)	35 (6.8%)	1 (0.8%)
Field of Work			
Musculoskeletal	588 (92.0%)	460 (90.0%)	128 (100%)
Geriatric	27 (4.2%)	27 (5.3%)	0 (0.0%)
Neurologic	22 (3.4%)	22 (4.3%)	0 (0.0%)
Cardiac, respiratory, paediatric	2 (0.3%)	2 (0.4%)	0 (0.0%)
Type of Working			
Private Practice	433 (67.8%)	387 (75.7%)	46 (35.9%)
Employee	206 (32.2%)	124 (24.3%)	82 (64.1%)
Italian geographical zones			
North	382 (59.8%)	316 (61.8%)	66 (51.6%)
Centre	166 (26.0%)	128 (25.0%)	38 (29.7%)
South	91 (14.2%)	67 (13.1%)	24 (18.8%)
Patients with SIS Per Month			
< 5	272 (42.6%)	250 (48.9%)	22 (17.2%)
5–10	263 (41.2%)	224 (43.8%)	39 (30.5%)
11–20	61 (9.5%)	27 (5.3%)	34 (26.6%)
> 20	43 (6.7%)	10 (2.0%)	33 (25.8%)

*Abbreviation*: *OMPTs* Orthopaedic physical therapist, *SIS* Subacromial Impingement Syndrome

Opinions on both diagnostic process (clinical examination, imaging screening), management of treatment (physiotherapy and medical treatment) and outcome measurement were collected. The detailed percentages for each respondent’s answers for both OMPTs and orthopaedic surgeons, Odds Ratio values comparing the correct answer to the incorrect ones (based on the adherence of the answers to the available literature) and *p*-values are reported in Table 2.

**Table 2:** Analytic Responses

Questions	Samples			OMPTs		Orthopaedic surgeons		***p***-value
	Total (***n*** = 639)	OMPTs (***n*** = 511)	Orthopaedic surgeons (***n*** = 128)	Odds	CI 95%	Odds	CI 95%	
1- In the patient suffering from painful shoulder:				0.3	0.2 to 0.6	2.7	1.6 to 4.8	
**The combination of multiple tests has been shown to provide better accuracy**	573 (89.7%)	470 (92.0%)	103 (80.5%)					< 0.001
The use of single and pathology-specific tests is recommended	41 (6.4%)	24 (4.7%)	17 (13.3%)					
Tests have been shown to detect the structure that generates the symptoms	15 (2.3%)	10 (2.0%)	5 (3.9%)					
The tests have all been shown to have high specificity	10 (1.6%)	7 (1.4%)	3 (2.3%)					
2- Among the diagnostic tests which would appear to have a higher diagnostic utility, in particular to confirm the pathology if the test is positive:				0.2	0.1 to 0.4	5.3	2.3 to 12.4	
Hawkins-Kennedy test (90 ° flexion of the arm with internal rotation)	221 (34.6%)	144 (28.2%)	77 (60.2%)					< 0.001
Empty can (abduction on the scapular plane at 90 ° in internal rotation)	171 (26.8%)	159 (31.1%)	12 (9.4%)					
Neer sign (complete flexion of the arm in internal rotation)	135 (21.1%)	102 (20.0%)	33 (25.8%)					
**Lift-off (arm in back position with back of the hand on the lumbar spine and active intra-rotation)**	112 (17.5%)	106 (20.7%)	6 (4.7%)					
3- Using diagnostic tests for patients with painful shoulder, clinical applicability is obstaculated by:				1.4	0.9 to 2.2	0.7	0.5 to 1.1	
A disagreement on the interpretation of the results	396 (62.0%)	336 (65.8%)	60 (46.9%)					0.141
**An extreme diversity in execution**	153 (23.9%)	116 (22.7%)	37 (28.9%)					
A great variability in the nomenclature	49 (7.7%)	34 (6.7%)	15 (11.7%)					
A great variability of the professional figures who administered them	41 (6.4%)	25 (4.9%)	16 (12.5%)					
4- The diagnosis of the rotator cuff pathology should be based on:				0.1	0.1 to 0.1	10.7	6.9 to 16.6	
**History of the patient and physical examination**	464 (72.6%)	424 (83.0%)	40 (31.3%)					< 0.001
Physical examination and bioimaging (Rx, Magnetic Resonance, Ultrasound)	145 (22.7%)	70 (13.7%)	75 (58.6%)					
Biomaging (Rx, Magnetic Resonance, Ultrasound)	20 (3.1%)	8 (1.6%)	12 (9.4%)					
History of the patient	10 (1.6%)	9 (1.8%)	1 (0.8%)					
5- Orthopaedic tests used to diagnostic the SIS:				0.4	0.3 to 0.7	2.2	1.3 to 3.5	
**They identify as healthy those who do not really present the disease**	195 (30.5%)	171 (33.5%)	24 (18.8%)					0.001
They identify the patients who actually present the disease as sick	114 (17.8%)	82 (16.0%)	32 (25.0%)					
They identify people who are really sick as sick and at the same time identify people who do not really present the disease as healthy	165 (25.8%)	103 (20.2%)	62 (48.4%)					
They do not identify patients who actually present the disease as sick	165 (25.8%)	155 (30.3%)	10 (7.8%)					
6- In the detection of total or partial injuries to the rotator cuff:				0.3	0.2 to 0.4	3.5	2.3 to 5.3	
**US was the most suitable method in terms of cost / effectiveness ratio**	359 (56.2%)	318 (62.2%)	41 (32%)					< 0.001
Magnetic resonance imaging (MRI) is the most suitable method in terms of cost / effectiveness ratio	240 (37,6%)	158 (30.9%	82 (64.1%)					
It is better not to use the ultrasound (US)	20 (3.1%)	15 (2.9%)	5 (3.9%)					
MRI is lower in terms of specificity	20 (3.1%)	20 (3.9%)	0 (0.0%)					
7- In the detection of full thickness rotator cuff tear, how have the following methods revealed their ability to frame the patients as healthy (not really having the pathology):				1.2	0.8 to 1.8	0.8	0.5 to 1.2	
**MRA better than MRI and US**	329 (51.5%)	240 (47.0%)	89 (69.5%)					0.389
US, MRI and Magnetic Resonance Arthrography (MRA) with equal efficacy	193 (30.2%)	167 (32.7%)	26 (20.3%)					
US better than MRI, better than MRA	70 (11.0%)	60 (11.7%)	10 (7.8%)					
US better than MRA, better than MRI	47 (7.4%)	44 (8.6%)	3 (2.3%)					
8- In the detection of partial thickness rotator cuff tears:				1.6	1.1 to 2.3	0.6	0.4 to 0.9	
**US and MRI have revealed high ability to frame those who actually presented with pathology as sick**	248 (38.8%)	187 (36.6%)	61 (47.7%)					0.022
MRI and MRA have the same ability to frame those who do not really have the disease as healthy subjects	171 (26.8%)	140 (27.4%)	31 (24.2%)					
MRA and US have revealed low ability to frame those subjects who really had the condition, such as sick people	136 (21.3%)	123 (24.1%)	13 (10.2%)					
MRI has detected ability to frame subjects who did not actually present pathology as healthy subjects in 100% of cases	84 (13.1%)	61 (11.9%)	23 (18.0%)					
9- For the detection of Supraspinatus tendon partial tears:				1.2	0.8 to 1.9	0.0	0.0 to 0.1	
MRA is better than MRI in framing patients who actually present the disease as sick	270 (42.3%)	198 (38.7%)	72 (56.3%)					0.393
**MRA is better than MRI in framing patients who do not really present the disease as healthy**	161 (25.2%)	125 (24.5%)	36 (28.1%)					
MRI appears to have poor ability to frame those who really do not have the disease as sick	111 (17.4%)	103 (20.2%)	8 (6.3%)					
MRA has shown poor diagnostic accuracy	97 (15.2%)	85 (16.6%)	12 (9.4%)					
10- What is the best treatment choice for the management of patients with SIS?				0.0	0.0 to 0.1	24.6	9.2 to 65.7	
**Physiotherapic treatment**	609 (95.3%)	506 (99.0%)	103 (80.5%)					< 0.001
Surgical treatment	13 (2.0%)	1 (0.2%)	12 (9.4%)					
Drugs	10 (1.6%)	3 (0.6%)	7 (5.5%)					
Physical therapy (diathermy, laser ...)	7 (1.1%)	1 (0.2%)	6 (4.7%)					
11- What is the main goal of the therapeutic exercise with this type of patient?				2.9	1.9 to 4.3	0.3	0.2 to 0.5	
**Educate and reassure the patient**	294 (46.0%)	285 (55.8%)	9 (7.0%)					< 0.001
Pain reduction	212 (33.2%)	144 (28.2%)	68 (53.1%)					
Recovery of functional limitation	91 (14.2%)	62 (12.1%)	29 (22.7%)					
Solving the mechanical problem	42 (6.6%)	20 (3.9%)	22 (17.2%)					
12- Which treatment do you believe should be used first with this type of patient?				0.1	0.0 to 0.1	16.4	8.0 to 33.6	
**Physiotherapy conservative treatment**	594 (93.0%)	500 (97.8%)	94 (73.4%)					< 0.001
Pharmacological treatment	40 (6.3%)	10 (2.0%)	30 (23.4%)					
Absolute rest	3 (0.5%)	1 (0.2%)	2 (1.6%)					
Surgical treatment	2 (0.3%)	0 (0.0%)	2 (1.6%)					
13- According to the current literature, with what type of treatment do patients with SIS really obtain better results in the short period?				0.4	0.3 to 0.6	2.5	1.7 to 3.8	
**Conservative treatment**	482 (75.4%)	405 (79.3%)	77 (60.2%)					< 0.001
Pharmacological treatment	125 (19.6%)	82 (16.0%)	43 (33.6%)					
Surgical treatment	24 (3.8%)	18 (3.5%)	6 (4.7%)					
Absolute rest	8 (1.3%)	6 (1.2%)	2 (1.6%)					
14- Regarding conservative treatment, which mode do you consider preferable to obtain a better functionality?				0.4	0.3 to 0.7	2.4	1.5 to 3.8	
**Therapeutic exercise**	531 (83,1%)	439 (85.9%)	92 (71.9%)					< 0.001
Manual therapy	84 (13.1%)	69 (13.5%)	15 (11.7%)					
Physical therapies	15 (2.3%)	1 (0.2%)	14 (10.9%)					
Stretching	9 (1.4%)	2 (0.4%)	7 (5.5%)					
15- The focus of the exercise therapy, should be:				3.1	2.0 to 4.9	0.3	0.2 to 0.5	
No one in particular	308 (48,2%)	296 (57.9%)	12 (9.4%)					< 0.001
Scapulo-thoracic dyskinesia	177 (27,7%)	122 (23.9%)	55 (43.0%)					
**Rotator cuff**	112 (17,5%)	70 (13.7%)	42 (32.8%)					
Capsular stretching	42 (6,6%)	23 (4.5%)	19 (14.8%)					
16- The exercise should be administered:				0.2	0.1 to 0.4	4.1	2.6 to 6.4	
**In a few different ways (few exercises)**	308 (48,2%)	279 (54.6%)	29 (22.7%)					< 0.001
In the absence of pain	208 (32,6%)	123 (24.1%)	85 (66.4%)					
With pain	69 (10,8%)	67 (13.1%)	2 (1.6%)					
With high repetitions	54 (8,5%)	42 (8.2%)	12 (9.4%)					
17- Which manual therapy strategies, among the following, do you consider preferable to obtain a better functionality in patients with SIS?				0.4	0.3 to 0.6	2.4	1.6 to 3.7	
**Soft tissue techniques (trigger point, muscle energy etc.)**	302 (47.3%)	265 (51.5%)	39 (30.5%)					< 0.001
Mobilization	284 (44.4%)	228 (44.6%)	56 (43.8%)					
Neurodynamic techniques	27 (4.2%)	5 (1.0%)	22 (17.2%)					
Manipulations	26 (4.1%)	15 (2.9%)	11 (8.6%)					
18- Which pharmacological strategies, among the following, do you consider preferable to obtain a better functionality in patients with SIS?				0.4	0.2 to 0.9	2.4	1.2 to 4.9	
Nonsteroidal anti-inflammatory drugs	301 (47.1%)	260 (50.9%)	41 (32.0%)					0.015
Corticosteroid injection	202 (31.6%)	125 (24.5%)	77 (60.2%)					
**Anesthetics – Painkillers**	87 (13.6%)	78 (15.3%)	9 (7.0%)					
Placebo (e.g., inert pill)	49 (7.7%)	48 (9.4%)	1 (0.8%)					
19- Which surgical procedure, among the following, do you consider preferable to obtain a better functionality in patients with SIS?				6.0	4.0 to 9.1	0.2	0.1 to 0.2	
Arthroscopic subacromial decompression	360 (56.3%)	321 (62.8%)	39 (30.5%)					< 0.001
**Arthroscopic acromioplastic and bursectomy**	195 (30.5%)	114 (22.3%)	81 (63.3%)					
Radiofrequency therapy or injections of platelet gel and leukocytes	65 (10.2%)	59 (11.5%)	6 (4.7%)					
Open subacromial decompression	19 (3.0%)	17 (3.3%)	2 (1.6%)					
20- How can you best measure the effectiveness of a treatment in a patient with SIS?				0.5	0.4 to 0.8	1.8	1.2 to 2.8	
**With validated multidimensional scales**	457 (71.5%)	379 (74.2%)	78 (60.9%)					0.003
With scales on functionality	121 (18.9%)	95 (18.6%)	26 (20.3%)					
With an interview	35 (5.5%)	25 (4.9%)	10 (7.8%)					
With scales for pain	26 (4.1%)	12 (2.3%)	14 (10.9%)					

*Abbreviation***:** *OMPTs* Orthopaedic Manipulative Physical Therapists, *SIS* Subacromial Impingement Syndrome, *US* Ultrasound, *MRI* Magnetic Resonance Imaging, *MRA* Magnetic Resonance Arthrography, *CI 95%* Confidence Interval, 95%

Correct answers are reported in bold

### Clinical examination

Regarding the administration of special tests for patients with SIS, respectively 92% (*n* = 470) of the OMPTs and 80.5% (*n* = 103) of orthopaedic surgeons stated that in patients with SIS, a combination of diagnostic tests produced better accuracy (e.g., the ability to differentiate correctly between patient and healthy subjects) [3] (*p* < 0.001 between groups).

Regarding the specificity of diagnostic tests, most OMPTs answered that the Empty can test had the highest specificity (*n* = 159; 31.1%), and the Neer sign had the least specificity (*n* = 102; 20.0%). In the sample of orthopaedic surgeons, most answered that the Hawkins-Kennedy test has the highest specificity (*n* = 77; 60.2%) while the “Lift off test” had the lowest specificity (*n* = 6; 4.7%).

In regard to the difficulty to systematically apply diagnostic tests in patients with SIS, the majority of respondents in both samples (OMPTs: *n* = 336; 65.8%; orthoapedic surgeons: *n* = 60; 46.9%) stated that there are issues in interpreting the results of a study.

In terms of diagnosing patients with SIS, the majority of OMPTs stated that diagnosis should be reached by recording information from both the history and clinical examination (*n* = 424; 83.0%), while most orthopaedic surgeons responded with clinical examination and imaging (*n* = 75; 58.6%).

In regard to the question of what clinical tests are used for diagnosing SIS, the majority of OMPTs stated that diagnostic tests are able to correctly detect healthy subjects (*n* = 171; 33.5%). Most orthopaedic surgeons answered that clinical tests are able to diagnose those with SIS and can also identify those without SIS (*n* = 62; 48.4%).

Detailed respondents’ answers on clinical examination are described in Table 2 (Questions number 1–5).

### Imaging screening

Regarding the most reliable techniques for detecting total or partial tears of the rotator cuff between USI, RMI, and RMA, the most common OMPT choice identified USI as the most favorable method in terms of cost-efficacy for investigating the total or partial tear (*n* = 318; 62.2%), while most orthopaedic surgeons answered RMI (*n* = 82; 64.1%).

Regarding sensitivity of the imaging techniques for detecting full thickness tears of the rotator cuff, the majority of OMPTs (*n* = 240; 47.0%) and orthopaedic surgeons (*n* = 89; 69.5%) stated that RMA is more capable of detecting full thickness tear of the rotator cuff than USI or RMI.

Regarding the ability of the imaging techniques in diagnosing partial thickness rotator cuff tears, the majority of OMPTs (*n* = 187; 36.6%) and orthopaedic surgeons (*n* = 61; 47.7%) stated that USI and RMI have high specificity (defining the ability to detect patients with partial thickness rotator cuff tears).

Regarding diagnostic imaging and partial thickness tears specific to the Supraspinatus tendon, the majority of the OMPTs (*n* = 198; 38.7%) and orthopaedic surgeons (*n* = 72; 56.3%) stated that MRA is more specific compared to RMI.

Detailed respondents’ answers on imaging screening are shown in Table 2 (Questions number 6–9).

### Strategies of management

Regarding strategies of management for patients with SIS, most respondents for both the samples answered physiotherapy treatment (OMPTs: *n* = 506; 99.0%; orthopaedic surgeons: *n* = 103; 80.5%).

In terms of what should be done at the first treatment for patients with SIS, most OMPTs (*n* = 500; 97.8%) and orthopaedic surgeons (*n* = 94; 73.4%) answered physiotherapy/conservative treatment.

In terms of what treatment has more short-term beneficial effect, most respondents answered conservative treatment (OMPTs: *n* = 405; 79.3%; orthopaedic surgeons: *n* = 77; 60.2%).

Detailed respondents’ answers on management strategies are described in Table 2 (Questions number 10–13).

### Strategies of physiotherapy treatment

Regarding which conservative strategy is more likely to recover function, for both samples most respondents answered therapeutic exercise (OMPTs: *n* = 439; 85.9%; orthopaedic surgeons: *n* = 92; 71.9%).

Regarding the main objective of therapeutic exercise in patients with SIS, the majority of OMPTs (*n* = 285; 55.8%) stated that education and reassurance of the patient should be the main objective, while the majority of orthopaedic surgeons (*n* = 68; 53.1%) identified pain reduction as the main goal.

Regarding the focus of therapeutic exercise, most OMPTs (*n* = 296; 57.9%) answered that exercise should not be targeted on a particular structure. Most orthopaedic surgeons (*n* = 55; 43.0%) answered that exercise should focus on scapular dyskinesis.

Regarding the administration of therapeutic exercise, most OMPTs (*n* = 279; 54.6%) answered that very few exercises should be administered in patients with SIS, while most orthopaedic surgeons (*n* = 85; 66.4%) responded that exercises should be pain-free.

Concerning the manual therapy modality that is more likely to promote functional recovery, most OMPTs (*n* = 265; 51.5%) answered techniques should target soft tissue; while most orthopaedic surgeons (*n* = 56; 43.8%) answered that mobilizations are more likely to promote functional recovery.

Detailed respondents’ answers on physiotherapy strategies are shown in Table 2 (Questions number 14–17).

### Medical treatment

Regarding which pharmacologic strategies are more likely to promote recovery of function, the majority of OMPTs (*n* = 260; 50.9%) felt that NSAIDs are more likely to promote recovery of function, while orthopaedic surgeons (*n* = 77; 60.2%) felt that corticosteroid injections were the answer.

In terms of the best surgical treatment for recovering function, OMPTs (*n* = 321; 62.8%) identified arthroscopic subacromial decompression, while orthopaedic surgeons (*n* = 81; 63.3%) chose arthroscopic bursectomy and acromioplasty.

Detailed respondents’ answers on medical treatment are described in Table 2 (Questions number 18 and 19).

### Collection of outcome measures

Regarding the most suitable outcome measures for patients with SIS, both samples of respondents agreed that PROs (patient reported outcomes) are the best outcome measures (OMPTs: *n* = 379; 74.2%; orthopaedic surgeons: *n* = 78; 60.9%) (*p* = 0.003).

Detailed respondents’ answers on outcome measures collection are shown in Table 2 (Question number 20).

## Discussion

To our knowledge, this is the first investigation regarding the adherence to the evidence in the assessment and treatment of patients with SIS performed among Italian clinicians. Our main finding indicates that OMPTs and orthopaedic surgeons choose different strategies in assessing and treating patients with SIS.

In accordance with evidence-based practice [16, 60], OMPTs attribute more value to the clinical examination and to the anamnesis in patients with SIS. Moreover, OMPTs were aware of the limitations of clinical tests and the valuable role that anamnesis and history provide in evaluating patients with SIS. These differences could be due to a higher predisposition in OMPTs to focus on function rather than a structural approach [39] compared to orthopaedic surgeons. This focus on function may have enhanced their use of the evidence, avoiding reliance on determining the structure that is causing the pain from SIS [47, 52].

In terms of imaging to identify partial or massive rotator cuff tears, our results (odds ratio and percentage) seem to be in favor of the orhopaedic surgeons, albeit by a non -statistically significant margin. However, in the current study, both categories of clinicians answered heterogeneously, highlighting the different findings of various studies on this topic [44]. One possible explanation could be that orthopaedic surgeons consider imaging as a key diagnostic factor due to its ability and competence in detecting structural failure, as well as for its utility in implementing the outcome in their practice [11, 22]. OMPTs in the current study correctly recognized USI as the best tool (in terms of cost/benefit ratio) [17] in detecting partial and massive rotator cuff tears. Even if imaging is increasingly used for assessment of patients with SP, the correlation between structural failures and pain and its role in informing management remain unknown, leaving the interpretation of imaging findings as part of a wider construct of assessment in SIS [69].

From a therapeutic point of view, our results have revealed the different core competence of these two categories of clinicians. OMPTs possess knowledge in identifying the goals, priority of assessment tools and different rehabilitation modalities such as exercise or manual therapy. To date, therapeutic exercise appears to be the best conservative treatment in the management of SIS in terms of reduction of pain and improvement of function [35]. Still, there is no agreement in the literature about the parameters of the exercise such as number of repetitions, optimal load, typology of contraction or modality (e.g. supervised or not) [2]. Both OMPTs and orthopaedic surgeons identified exercise as the best conservative strategy. Furthermore, orthopaedic surgeons correctly identified the right purpose of exercises used in rehabilitation. Orthopaedic surgeons (68%) were more competent than OMPTs (22%) in detecting the best surgical approaches when needed. Unexpectedly OMPTs were aware of the most effective pharmacological treatments (in terms of anaesthetics and painkillers) [18] compared to orthopaedic surgeons (15% vs 7%). Both OMPTs and orthopaedic surgeons indicated the use of NSAID or corticosteroids as high (respectively ~ 75% and ~ 92%). This finding is consistent with the fact that NSAID and corticosteroids are the most studied drugs in the literature for patients with SIS [66]. It also suggests that both OMPTs and orthopaedic surgeons consider inflammation as the main source of the pain in patients with SIS [18, 73]. This approach is limited to the biomedical model, not considering the biopsycosocial nature of the patient’s symptoms of pain such as fear, self-efficacy and expectations [10, 13, 21, 49, 63].

The interpretation of data regarding PROs suggests that the concept of multidimensionality behind the patient’s pain and health in general is not yet understood. Even if OMPTs are more inclined than orthopedic surgeons (74% vs 61%) to use validated multidimensional scales, there is no statistically significant difference in relation to the choice of what instrument is used.

An interesting element emerges from the results of questions regarding the choices of therapeutic strategies for the patient. There is strong evidence suggesting that physiotherapy treatment is the best conservative strategy initially and for short-term effects [4, 12, 65]. OMPTs from the current study were more aware than orthopaedic surgeons about this, with a statistically significant difference in all three questions dedicated to this topic. Indeed, between 23 and 33% of orthopaedic surgeons consider pharmacological treatment as the first and the most effective therapeutic intervention to advise patients with SIS for short-term relief of pain. As a possible source of explanation Italian OMPTs were trained by their University to apply conservative strategies for the management of the patients’ complaints without using drugs or prescripting images (e.g., US, MRI, MRA) [27]. Contrarily, orthopaedic surgeons were educated to use drugs and to perform surgery during their University training, thus limiting their knowledge about physiotherapy and rehabilitation [40]. The results from the current study agree with other surveys on rotator cuff disorders performed in different European countries such as Italy [8], United Kingdom [9], Belgium and Netherlands [58].

A simplistic understanding of this data may suggest that OMPTs are better than orthopaedic surgeons when it comes to counselling a patient on optimum management. However, it should be taken into account that the population of OMPTs chosen were specialists (professionals specialized in musculoskeletal disorders rehabilitation). In addition, there are some differences between OMPTs and orthopaedic surgeons in terms of age (under 40 age: OMPTs 85%; orthopaedics surgeons 35%), experience (over 10 years OMPTs 77%; orthopaedics surgeons 30%) but also in setting (private practice: OMPTs 76%; orthopaedics 36%). These differences may have influenced the outcomes of the current study as not all OMPTs work in a direct access environment and are specialized in musculoskeletal disorders rehabilitation even if these OMPTs have less experience than the orthopaedic surgeons in the current study.

### Limits

Limitations in every study exist and attempts are made to clear up as many as possible a priori in designing a study. Limits with submitting a survey are common as one does not know who will respond and how the questions will be interpreted by the respondent.

The first limitation within the current study is that there is not homogeneity of the two samples chosen for the survey which effects the generalization of the results. Thus, the results of this survey are specific to those who have responded to the survey at that point in time and the interpretation of the respondent answers may not be entirely accurate.

Sample characteristics could be the second limitation of this survey. Five-hundred-and-eleven OMPTs participated in our study from an overall Italian OMPTs population of 1570. In the current study, the authors chose to use OMPTs specialized in musculoskeletal management and not all physiotherapists in Italy are specialized in this area. Our sample represents 31% of Italian OMPTs which could be debated as being representative of all physiotherapists in Italy. It would have been ideal to have more but in conducting a survey it is hard to determine how many will respond. Regarding the number of males and young people in this sample, actually we think that this could be representative of the OMPT population in Italy. In fact most OMPTs in Italy are under 35 years (82%) and are male (70%) according to a previous survey on Italian OMPTs [61].

It also would have been ideal to have had the same number of orthopaedic surgeons answering the survey as physiotherapists but this was not the case. The sample of orthopaedic surgeons was not representative as it is equivalent to 4% of the overall population (128 out of 3196 of orthopaedic surgeons registered in the association responded to the survey). Future studies should attempt to have comparable sizes of both groups and homogeneous samples to compare and contrast the two groups and their responses. Perhaps future studies could consider investigating competency and adherence to efficacy testing across both populations, as well as how both populations assess and treat other common causes of shoulder pain for patients that present to both clinical practices (for example shoulder instability and adhesive capsulitis). Furthermore, future studies should be conducted in extra European countries because all surveys present in the literature investigate European physiotherapist samples.

## Conclusions

In summary, OMPTs and orthopaedic surgeons approached patients with SIS somewhat differently on the initial visit for assessment and treatment. In this survey, Italian OMPTs specialized in musculoskeletal rehabilitation appear to be appropriate in planning and managing clinical examination and therapeutic strategies to use with patients with SIS.

## Data Availability

The datasets used and/or analysed during the current study are available from the corresponding author on reasonable request.

## Appendix

### The online survey

#### Section (A) – Demographic questions

1. ***Occupation***

- Orthopaedic surgeon
- OMPT

2- ***Gender***

- Male
- Female

3- ***Age (Years)***

- < 30
- 31–40
- 41–50
- > 50

4- ***Years of clinical practice***

- < 5
- 5–10
- 11–15
- > 15

5- ***Workplace***

- Hospital
- Residential care (nursing home)
- Private practice

6- ***Field of work***

- geriatric
- neurologic
- musculoskeletal
- cardiologic, respiratory, pediatric

7- ***Type of work***

- employee
- private practice

8- ***Italian geographical Zones***

- North of Italy
- Center of Italy
- South of Italy

9- ***Patients with SIS Per Month***

- < 5
- 6–10
- 11–20
- > 20

#### Section (B) – Technical questions

10- ***In the patient suffering from painful shoulder:***

- The combination of multiple tests has been shown to provide better accuracy;
- The use of single and pathology-specific tests is recommended;
- Tests have been shown to detect the structure that generates the symptoms;
- The tests have all been shown to have high specificity;

11- ***Among the diagnostic tests which would appear to have a higher diagnostic utility, in particular to confirm the pathology if the test is positive:***

- Hawkins-Kennedy test (90 ° flexion of the arm with internal rotation)
- Empty can (abduction on the scapular plane at 90 ° in internal rotation)
- Neer sign (complete flexion of the arm in internal rotation)
- Lift-off (arm in back position with back of the hand on the lumbar spine and active intra-rotation)

12- ***Using diagnostic tests for patients with painful shoulder, clinical applicability is obstaculated by:***

- A disagreement on the interpretation of the results
- An extreme diversity in execution
- A great variability in the nomenclature
- A great variability of the professional figures who administered them

13- ***The diagnosis of the rotator cuff pathology should be based on:***

- History of the patient and physical examination
- Physical examination and bioimaging (Rx, Magnetic Resonance, Ultrasound)
- Bioimaging (Rx, Magnetic Resonance, Ultrasound)
- History of the patient

14- ***Orthopaedic tests used to diagnosticate the SIS:***

- They identify as healthy those who do not really present the disease
- They identify the patients who actually present the disease as sick
- They identify people who are really sick as sick and at the same time identify people who do not really present the disease as healthy
- They do not identify patients who actually present the disease as sick

15- ***In the detection of total or partial injuries to the rotator cuff:***

- US was the most suitable method in terms of cost / effectiveness ratio
- Magnetic resonance imaging (MRI) is the most suitable method in terms of cost / effectiveness ratio
- It is better not to use ultrasound (US)
- MRI is lower in terms of specificity

16- ***In the detection of full thickness rotator cuff tear, how have the following methods revealed their ability to frame the patients as healthy (not really having the pathology):***

- MRA (Magnetic Resonance Arthrography) better than MRI and US
- US, MRI and MRA with equal efficacy
- US better than MRI, better than MRA
- US better than MRA, better than MRI

17- ***In the detection of partial thickness rotator cuff tears:***

- US and MRI have revealed high ability to frame those who actually presented with pathology as sick
- MRI and MRA have the same ability to frame those who do not really have the disease as healthy subjects
- MRA and US have revealed low ability to frame those subjects who really had the condition, such as sick people
- MRI has detected ability to frame subjects who did not actually present pathology as healthy subjects in 100% of cases

18- ***For the detection of Supraspinatus tendon partial tears:***

- MRA is better than MRI in framing patients who actually present the disease as sick
- MRA is better than MRI in framing patients who do not really present the disease as healthy
- MRI appears to have poor ability to frame those who really do not have the disease as sick
- MRA has shown poor diagnostic accuracy

19- ***What is the best treatment choice for the management of patients with SIS?***

- Physiotherapy treatment
- Surgical treatment
- Drugs
- Physical therapy (diathermy, laser ...)

20- ***What is the main goal of therapeutic exercise with this type of patients?***

- Educate and reassure the patient
- Pain reduction
- Recovery of functional limitation
- Solving the mechanical problem

21- ***Which treatment do you believe should be used first with this type of patient?***

- Conservative physiotherapy treatment
- Pharmacological treatment
- Absolute rest
- Surgical treatment

22- ***According to the current literature, with what type of treatment do patients with SIS really obtain better results in the short period?***

- Conservative treatment
- Pharmacological treatment
- Surgical treatment
- Absolute rest

23- ***Regarding to conservative treatment, which mode do you consider preferable to obtain better functionality?***

- Therapeutic exercise
- Manual therapy
- Physical therapies
- Stretching

24- ***The focus of the exercise therapy, should be:***

- Not one in particular
- Scapulo-thoracic dyskinesia
- Rotator cuff
- Capsular stretching

25- ***The exercise should be administered:***

- In a few different ways (few exercises)
- In the absence of pain
- With pain
- With high repetitions

26- ***Which manual therapy strategies, among the following, do you consider preferable to obtain a better functionality in patients with SIS?***

- Soft tissue techniques (trigger point, muscle energy etc.)
- Mobilization
- Neurodynamic techniques
- Manipulations (High Velocity Low Amplitude Thrust -HVLAT-)

27- ***Which pharmacological strategies, among the following, do you consider preferable to obtain a better functionality in patients with SIS?***

- Nonsteroidal anti-inflammatory drugs
- Corticosteroid injection
- Anesthetics - Painkillers
- Placebo (e.g. inert pill)

28- ***Which surgical procedure, among the following, do you consider preferable to obtain a better functionality in patients with SIS?***

- Arthroscopic subacromial decompression
- Arthroscopic acromioplastic and bursectomy
- Radiofrequency therapy or injections of platelet gel and leukocytes
- Open subacromial decompression

29- ***How can you best measure the effectiveness of a treatment in a patient with SIS?***

- With validated multidimensional scales
- With scales on functionality
- With an interview
- With scales for pain
